# Abundant Genetic Diversity of Yunling Cattle Based on Mitochondrial Genome

**DOI:** 10.3390/ani9090641

**Published:** 2019-09-02

**Authors:** Xiaoting Xia, Kaixing Qu, Fangyu Li, Peng Jia, Qiuming Chen, Ningbo Chen, Jicai Zhang, Hong Chen, Bizhi Huang, Chuzhao Lei

**Affiliations:** 1 Key Laboratory of Animal Genetics, Breeding and Reproduction of Shaanxi Province, College of Animal Science and Technology, Northwest A&F University, Yangling 712100, Shaanxi, China; 2 Yunnan Academy of Grassland and Animal Science, Kunming 650212, Yunnan, China

**Keywords:** Yunling cattle, mtDNA genome, haplogroup, maternal origin

## Abstract

**Simple Summary:**

Mitochondrial DNA (mtDNA) analysis is a critical tool in assessing the maternal origin, phylogeny and population structure of domestic animals. Yunling cattle are a composite breed that was created by local Yunnan cattle breeds (as a maternal line) 30 years ago. It can be said that Yunling cattle represent important reservoirs of genetic diversity of local Yunnan cattle. Yunling cattle are characterized as a tropical/subtropical breed with fast growth, heat tolerance and parasite resistance, and can survive in a harsh environment and on low-quality roughage. Assessing the genetic characteristics of Yunling cattle is of particular importance for reasonable breeding strategies for Yunling cattle and the design of a local Yunnan cattle conservation program.

**Abstract:**

Yunling cattle are a composite beef cattle breed, combining Brahman (1/2), Murray Grey (1/4) and Local Yunnan cattle (1/4), that was developed in Yunnan, China in the 1980s. Understanding the genetic information of Yunling cattle is of great significance to the development of reasonable breeding strategies for this breed. In the present study, we assessed the current genetic status of Yunling cattle in Yunnan Province (China) by analyzing the variability of the whole mitochondrial genome of 129 individuals. Altogether, 129 sequences displayed 47 different haplotypes. The haplotype diversity and the average number of nucleotide differences were 0.964 and 128.074, respectively. Phylogenetic analyses classified Yunling cattle into seven haplogroups: T1, T2, T3, T4, T6, I1 and I2. Haplogroup I1 was found to be predominant (41.86%), followed by T3 (28.68%). Furthermore, we also identified a novel haplogroup, T6, and defined the sub-haplogroup I1a in Yunling cattle. According to the formation process of Yunling cattle (local Yunnan cattle as the maternal line), the high genetic diversities in the mitochondria of Yunling cattle could be due to the complex maternal origin of local Yunnan cattle. Further studies about local Yunnan breeds are necessary to determine the exact source of haplogroup T6 in Yunling cattle. Our results will be useful for the evaluation and effective management of Yunling cattle.

## 1. Introduction

Yunling cattle are a cultivated breed achieved by crossbreeding with Brahman (bull), Murray Grey (bull) and local Yunnan cattle (Wenshan and Zhaotong cattle as maternal lines) for more than 30 years [1,2]. In 1984, 87 Murray Grey cattle from Australia were imported into China to improve the production performance of local Yunnan cattle through hybridization. Subsequently, the Murray Grey × Yunnan cattle hybrid females were “graded up” using Brahman bulls, also imported from Australia. Thus, Yunling cattle are featured as a tropical/subtropical breed with the following characteristics: (1) enhanced growth; (2) heat tolerance; (3) resistance to parasitic infection; (4) ability to survive in harsh environments; (5) ability to survive on low-quality roughage [1].

Assessing the genetic characteristics of Yunling cattle is of particular importance for the development of a reasonable breeding strategy for Yunling cattle and the design of a local Yunnan cattle conservation program. The genetic diversity of Yunling cattle has previously been examined using a range of techniques, including karyotypic analysis [3], microsatellite DNA markers [1] and Y-chromosome polymorphisms (Y-SNPs and Y-STRs) [4]. Here, we present additional information on the genetic diversity of the Yunling breed obtained from the analysis of the mitochondrial DNA (mtDNA). Previous studies of mtDNA genetic diversity have revealed eight major mtDNA haplogroups in cattle, the taurine T, T1, T2, T3, T4 and T5 as well as the indicine I1 and I2 [5,6,7,8]. To date, most mtDNA studies have generally focused on the control region. However, using the entire mitochondrial genome sequence to study the genetic structure of animals can provide us with refined phylogenies of maternal lineages [7].

In this study, we sequenced the complete mtDNA of Yunling cattle along with that of Brahman cattle, a hybrid breed that was achieved by the repeated crossbreeding of local *Bos taurus* from the United States with imported zebu from India [9], for genetic diversities and phylogenetic analysis. The aim of our study was to determine the matrilineal genetic diversity and phylogenetic status of Yunling cattle.

## 2. Materials and Methods

### 2.1. Animal Sampling

We collected 129 Yunling cattle and 31 Brahman cattle (control) ear tissues for mtDNA analysis at Xiaoshao Farm of Yunnan Academy of Grassland and Animal Science, Kunming, Yunnan, China. To minimize the degree of relationship among individuals, animals were selected according to pedigree information. Genomic DNA was extracted by the standard phenol-chloroform method [10].

### 2.2. Illumina Sequencing and Reconstruction of Mitochondrial Genomes

Sequencing was performed at the Novogene Bioinformatics Technology Co., Ltd., Beijing, China. A total amount of 1 μg DNA per sample was used as input material for the DNA sample preparations. Sequencing libraries were generated using the NEBNext^®^ Ultra™ DNA Library Prep Kit for Illumina (NEB, USA) following the manufacturer’s recommendations, and index codes were added to attribute sequences to each sample. Briefly, the DNA sample was fragmented by sonication to a size of 350 bp, then DNA fragments were end-polished, A-tailed and ligated with the full-length adaptor for Illumina sequencing with further PCR amplification. Lastly, PCR products were purified (AMPure XP system (Beckman Coulter, Beverly, MA, USA)) and libraries were analyzed for size distribution by an Agilent 2100 Bioanalyzer (Agilent Technologies, Palo Alto, CA, USA) and quantified using real-time PCR. The clustering of the index-coded samples was performed on a cBot Cluster Generation System according to the manufacturer’s instructions. After cluster generation, the library preparations were sequenced on an Illumina HiSeq platform and paired-end reads were generated.

The Burrows-Wheeler Aligner BWA-MEM (v0.7.13-r1126) with default parameters was used to map the paired-end reads to the mitochondrion MT (GenBank: AY526085.1) from the latest reference genome (ARS-UCD1.2). For each sample, the reads showing the unique hit after removal of the duplicate were considered for subsequent analysis. The average depth-of-coverage was 619.08 X ranging from 95.94 to 1279.31 X (Table S1). BAM alignments were transformed to FASTQ files and then Mapping Iterative Assembler v 1.0 (MIA) was used to assemble a mtDNA consensus sequence. The basic idea of the MIA is to align DNA sequencing fragments (shotgun or targeted resequencing) to a reference, then call a consensus. Subsequently, the consensus is used as new reference and the process is repeated until convergence (https://github.com/mpieva/mapping-iterative-assembler).

### 2.3. Data Analysis

Measures of mitogenome sequence variation, including numbers of haplotypes and variable sites, gene (haplotype) diversity, nucleotide diversity (Pi) and the average number of nucleotide differences (k), were computed using the program DnaSP v 5.10 [11]. A Bayesian phylogenetic tree was constructed using MRBAYES 3.1.2 [12] based on the GTR + G + I Model. Markov Chain Monte Carlo (MCMC) analyses were run for 1 × 107 generations with a burn-in value of 1250. Trees were sampled every 2000 generations. A neighbor-joining tree was constructed in MEGA 5.0 [13], and the reliability of the tree topology was assessed by 1000 bootstrap replications. The median-joining network was constructed using NETWORK 5.0.1.1 [14]. The sequences have been deposited in GenBank under the accession numbers MN200779–MN200938.

## 3. Results

### 3.1. MtDNA Sequence Variation and Genetic Diversity

**Figure 1:**
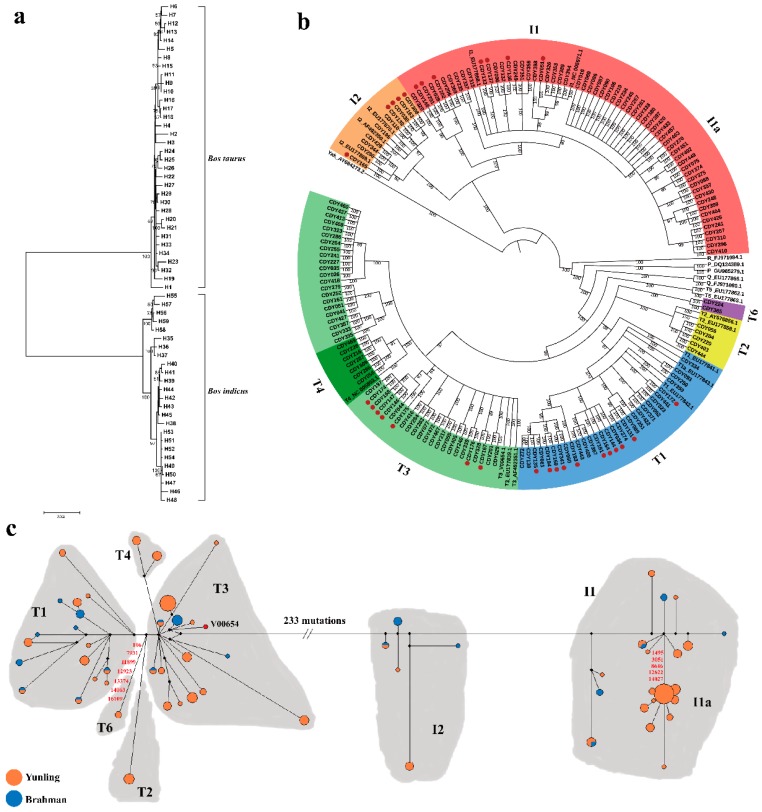
(**a**) A neighbor-joining (NJ) tree of 59 haplotypes in Yunling and Brahman cattle. The values on the branches were bootstrap supported based on 1000 replications. (**b**) A Bayesian phylogenetic tree of 129 Yunling and 31 Brahman (annotated with red dots) sequences, as well as 21 cattle reference sequences. This tree was rooted by using a *Bos grunniens* mitochondrial genome (accession no. AY684273). The values on the branches represent the posterior probabilities (percent). The GenBank accession numbers for the haplogroup references are shown in labels. (**c**) Median-joining network constructed from 160 sequences. Red numbers on the lines represent the new mutation sites relative to the bovine reference sequence (accession no. V00654) defined in this study. The areas of the circles are proportional to the haplotype frequencies. Black points are median vectors.

In the present study, we analyzed the sequence variation of the complete mitogenome of Yunling cattle to assess their mtDNA genetic variation and maternal origin. Analysis of mitogenome sequences (16,338 to 16,340 bp) revealed 405 variable sites (45 were singletons and 360 were parsimony informative) among the 129 samples, defining a total of 47 haplotypes (Table S2; Figure 1a). H53 was the most common haplotype with the highest frequency (13.19%, 17/129), followed by H32, which accounted for 9.30% (12/129) of all individuals. However, 20 haplotypes were observed only once. By analyzing the genetic structure, the average number of differences for Yunling was 128.074. The gene (haplotype) diversity of mtDNA sequences was 0.964 ± 0.007. Our results reveal the high genetic diversity of Yunling cattle. Similar high mtDNA diversity was also shown in Brahman cattle (haplotype diversity (Hd) ± SD = 0.959 ± 0.020). This may be related to the fact that both of these breeds are crossbred breeds. More detailed information on genetic diversity estimates, including the number of variable sites (S), the number of haplotypes (H) and nucleotide diversity (Pi ± SD), is given in Table 1.

The mitogenome shows considerable variation in diversity among different regions (Figure S1). The highest diversity was observed in the D-loop region (from np 15909 to np 16315) with a peak of Pi = 0.0548. These estimations are in accordance with previous data for other livestock related studies [15,16,17].

### 3.2. MtDNA Sequence Variation and Genetic Diversity

A Bayesian phylogenetic tree was constructed based on 160 complete mitogenomes (129 Yunling and 31 Brahman), combined with 21 representative sequences of mtDNA haplogroups (T1–T5, I1, I2, R, P and Q) retrieved from GenBank together with *Bos grunniens* mtDNA sequence (accession no. AY684273) as an outgroup (Figure 1b). The Bayesian tree divided all Yunling cattle sequences into two distinct haplogroups: *Bos taurus* T and *Bos indicus* I, respectively including 27 and 20 haplotypes.

Consequently, 54.26% (70/129) of Yunling cattle belonged to *Bos taurus* T-sub-haplogroups T1 (14.73%, 19/129), T2 (3.88%, 5/129), T3 (28.68%, 37/129) and T4 (5.43%, 7/129), as previously defined [5,6,7]. Interestingly, we also found two individuals unambiguously belonging to the T haplogroup, but representing an unknown divergent mitochondrial sub-haplogroup. The new sub-haplogroup was characterized by 12 mutations (106, 169, 2536, 7931, 9682, 11899, 12923, 13310, 13374, 14063, 16109 and 16255) relative to the bovine reference sequence (V00654) (H19; Figure 1c and Table S2), here named T6. The remaining 45.74% of Yunling mtDNA samples (59/129) were members of *Bos indicus* I haplogroup. Haplogroup I1 was the most frequent indicine haplogroup that occurred in 54 samples, followed by I2 occurring five times.

A median-joining network was also constructed to obtain further insights into the phylogenetic relationships of the Yunling breed. As expected, the analysis revealed two clusters in sampled cattle which were separated by 233 mutations (Figure 1c). Except for the novel haplogroup T6, mutation motifs for all taurine haplogroups are consistent with previously published diagnostic sites [7,18]. Within the *Bos indicus* lineage, the sub-haplogroup I1a was separated from I1 by five mutations (1495, 3051, 8646, 12622 and 14027) which showed a star-like pattern typical of domestic species, suggestive of a past population expansion as described by a previous study [19].

## 4. Discussion

Yunling cattle have a genetic background derived from crossbreeding with local Yunnan cows and Murray Grey bulls, and then “graded up” with Brahman bulls as a terminal sire. To date, at least six haplogroups (T1a, T2, T3, T4, I1 and I2) have been found in local Yunnan cattle [20,21]. Gou et al. (2015) also indicated that the maternal lineage of Yunnan cattle was the admixture of *Bos taurus* and *Bos indicus* [22]. In the current study, we confirmed a similar mtDNA topology with local Yunnan cattle, consisting of haplogroups of T2, T3, T4, I2 and predominantly I1 (41.86%). In addition, unexpectedly, we found a high incidence of African T1 matrilines in Yunling cattle with an overall frequency of 14.73%. In Asia, the T1 haplogroup has previously been identified only in a few cattle individuals from China, Korea and Japan [21,23,24,25]. Recently, Li et al. (2018) [20] detected a low frequency of T1a in Zhaotong cattle from Yunnan Province. Thus, we speculate that one of the sources of T1 for Yunling might from local Yunnan cattle, which is consistent with the breeding practices of this composite breed (with the Zhaotong breed as one of the matrilines). In other words, Yunling cattle might have retained the earlier mitochondrial haplogroups of Yunnan cattle. Another potential source of T1 for Yunling could be Brahman. A previous study reported Brahman cattle to be “phenotypically zebu with taurine mtDNA” [26]. Two mtDNA haplogroups, T1a and T3, have been defined in Brahman by the mtDNA D-loop region [26]. A similar high frequency of T1 was also found in our Brahman cattle samples (35.48%). The uncommon haplogroup T1 might have derived from Brahman females, which may be due to poorly managed farms or the free-grazing pattern in Yunling cattle farms.

In addition, from the identification of the novel haplogroup T6 that has not been described in modern cattle populations, it can be concluded that local Yunnan cattle have a unique and complex maternal origin that requires further study in the future.

## 5. Conclusions

In conclusion, our findings revealed a high level of maternal genetic diversity and two divergent origins in the Yunling breed. Furthermore, we detected a new haplogroup, T6, and confirmed the sub-haplogroup I1a in Yunling cattle. The detection of a high frequency of T1 indicates that the breeding practice of Yunling cattle may require further verification.

## Figures and Tables

**Table 1:** Genetic structure and diversity of Yunling and Brahman cattle.

Breed	Sample Size	Haplogroup							S	H	k	Hd ± SD	Pi ± SD
		T1	T2	T3	T4	T6	I1	I2					
Yunling	129	19	5	37	7	2	54	5	405	47	128.074	0.964 ± 0.007	0.00784 ± 0.00013
Brahman	31	11	0	8	0	0	7	5	312	20	123.394	0.959 ± 0.020	0.00756 ± 0.00067
Total	160	30	5	45	7	2	61	10	429	59	127.055	0.974 ± 0.005	0.00778 ± 0.00014

S: number of variable sites; H: number of haplotypes; k: the average number of differences; Hd: haplotype diversity; Pi: nucleotide diversity; SD: standard deviation.

## Supplementary Materials

The following are available online at https://www.mdpi.com/2076-2615/9/9/641/s1, Figure S1: Sequence variation within the Yunling cattle mitochondrial genome, Table S1: Overview of sample information and sequencing statistics, Table S2: Polymorphic sites for the mitochondrial genome haplotypes in Yunling and Brahman cattle.
