# Supplementary material for: Abundant Genetic Diversity of Yunling Cattle Based on Mitochondrial Genome
Source: Animals (Basel). 2019 Sep 2;9(9):641. doi: 10.3390/ani9090641 (PMC6769864; doi:10.3390/ani9090641)
Supplement: Supplementary file 1 [file animals-09-00641-s001.zip › supplementary - for proofreading/Supplementary Materials-for proofreading.docx]

Supplementary Materials: Abundant Genetic Diversity of Yunling Cattle Based on Mitochondrial Genome

Xiaoting Xia ^1^, Kaixing Qu ^2^, Fangyu Li ^1^, Peng Jia ^1^, Qiuming Chen ^1^, Ningbo Chen ^1^, Jicai Zhang ^2^, Hong Chen ^1^, Bizhi Huang ^2,^* and Chuzhao Lei ^1,^*

^1^ Key laboratory of Animal Genetics, Breeding and Reproduction of Shaanxi Province, College of Animal Science and Technology, Northwest A&F University, Yangling 712100, Shaanxi, China.

^2^ Yunnan Academy of Grassland and Animal Science, Kunming 650212, Yunnan, China.

***** Correspondence: hbz@ynbp.cn (B.H.); leichuzhao1118@126.com (C.L.)


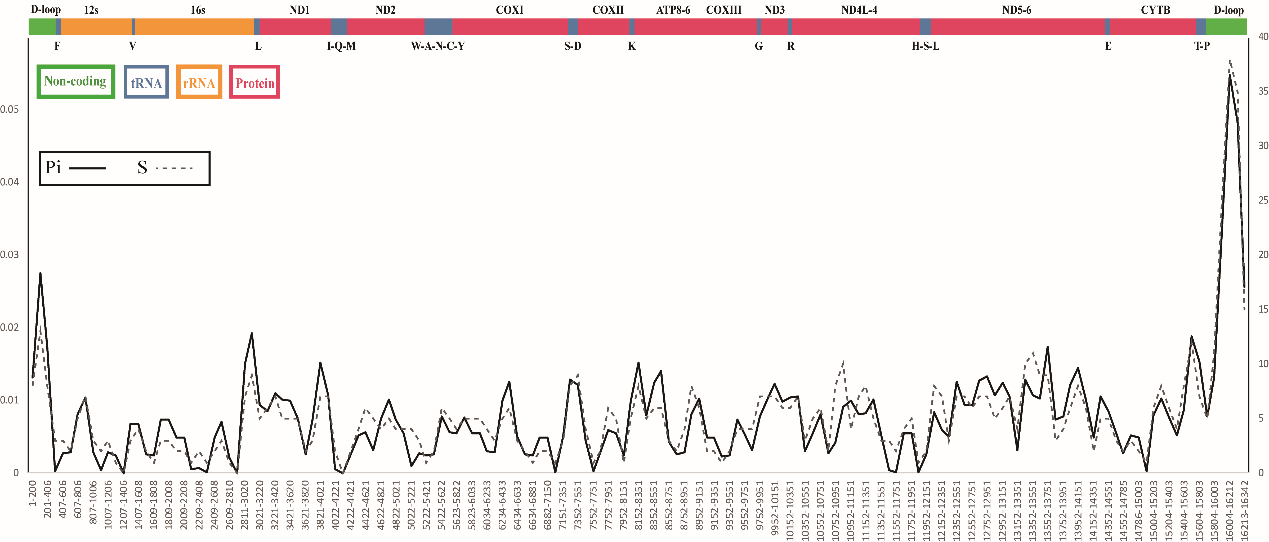


**Figure 1.** Sequence variation within Yunling cattle mitochondrial genome. The nucleotide diversity (continuous line) and the number of substitutions (dotted line) along the entire mtDNA were calculated by considering windows of 200 bps (step size = 100 bp) centered in the midpoint.
